# The Potential Value of Targeting Ferroptosis in Early Brain Injury After Acute CNS Disease

**DOI:** 10.3389/fnmol.2020.00110

**Published:** 2020-06-18

**Authors:** Junhui Chen, Yuhai Wang, Jiyun Wu, Jiaji Yang, Mingchang Li, Qianxue Chen

**Affiliations:** ^1^Department of Neurosurgery, Renmin Hospital of Wuhan University, Wuhan, China; ^2^Department of Neurosurgery, 904th Hospital of Joint Logistic Support Force of PLA, Wuxi Clinical College of Anhui Medical University, Wuxi, China; ^3^Department of Orthopedic, 904th Hospital of Joint Logistic Support Force of PLA, Wuxi Clinical College of Anhui Medical University, Wuxi, China

**Keywords:** ferroptosis, early brain injury, acute CNS disease, Nrf2, GPX4, ROS

## Abstract

Acute central nervous system (CNS) disease is very common and with high mortality. Many basic studies have confirmed the molecular mechanism of early brain injury (EBI) after acute CNS disease. Neuron death and dysfunction are important reasons for the neurological dysfunction in patients with acute CNS disease. Ferroptosis is a nonapoptotic form of cell death, the classical characteristic of which is based on the iron-dependent accumulation of toxic lipid reactive oxygen species. Previous studies have indicated that this mechanism is critical in the cell death events observed in many diseases, including cancer, tumor resistance, Alzheimer’s disease, Parkinson’s disease, stroke, and intracerebral hemorrhage (ICH). Ferroptosis may also play a very important role in EBI after acute CNS disease. Unresolved issues include the relationship between ferroptosis and other forms of cell death after acute CNS disease, the specific molecular mechanisms of EBI, the strategies to activate or inhibit ferroptosis to achieve desirable attenuation of EBI, and the need to find new molecular markers of ferroptosis that can be used to detect and study this process *in vivo* after acute CNS disease.

## Introduction

Acute central nervous system (CNS) disease is very common, including intracerebral hemorrhage (ICH), Spontaneous subarachnoid hemorrhage (SAH), traumatic brain injury (TBI), and ischemic stroke, etc. The latest report of the European Stroke Association shows that the incidence of SAH is approximately 9/100,000 (Feigin et al., 2009; Chen et al., 2020). The incidence in Asian countries is relatively high; for example, the incidence in Japan is approximately 22.7/100,000, which is similar to that in China (Chen et al., 2020). And the incidence of ICH, ischemic stroke, and TBI was even higher (Hanley et al., 2019; Jiang et al., 2019; Johnson et al., 2019). A foreign study reported a short-term prognosis in which the mortality of SAH was as high as 8.3–66.7%, with a mean median proportion of direct deaths before admission of 8.3%, and the percentage associated with good prognosis was only approximately 36–55% (Komotar et al., 2009). Most survivors of acute CNS disease face long-term postoperative cognitive and emotional impairments, loss of hearing and smell, and reduced quality of life (Nieuwkamp et al., 2009). The acute CNS disease treatment for patients is long, difficult, and expensive, and the huge treatment cost places a serious burden on the national economy. At the beginning of the 21st century, the average first-year hospitalization cost for a SAH patient with an aneurysm exceeded $65,000 in the United States (Passier et al., 2010), and the UK government paid more than 510 million pounds for the treatment of aneurysm-induced SAH in patients (Qureshi et al., 2007).

The mechanism of the acute CNS disease pathological process is extremely complex. Hemoglobin and its decomposition products are important materials in hemorrhage disease. After SAH/ICH-induced membrane permeabilization, red blood cells release large amounts of blood into the subarachnoid space, parenchyma or the brain, and then, the red blood cells, undergo fast pyrolysis, with hemoglobin and its breakdown products playing roles in neurotoxicity through various channels. Secondary brain damage has also been confirmed in a large number of animal experiments and supported by data from cell experiments (Chen et al., 2014). Although many basic studies have confirmed the molecular mechanism of early brain injury (EBI) after acute CNS disease and a large number of researchers have been recently focused on developing drugs based on anti-vasospasm, anti-apoptosis, and anti-inflammation mechanisms, most of the drugs have been confirmed to be ineffective in the treatment of EBI after acute CNS disease, as shown by multicenter randomized control trials (Macdonald, 2014). Therefore, are other molecular mechanisms involved after acute CNS disease, such as those related to a neuronal injury? Neurons are an important functional unit of the central nervous system. Neuron death and dysfunction are important reasons for neurological dysfunction in patients with acute CNS disease. Therefore, research on the mechanism of neuronal death and dysfunction has been one of the foci and hot topics in life science, neuroscience, medicine, and pharmacy fields. Traditional studies have confirmed that the main causes of neuronal death are necrosis, apoptosis, autophagy, pyroptosis, etc. Neuronal necrosis is considered a process of passive cell death, characterized by cell membrane rupture and cytoplasmic disintegration, neither of which can be regulated. However, apoptosis, autophagy, and pyroptosis are neuronal active death processes. Each process consumes energy and is regulated by cell signaling pathways, which is the basis for this type of death being called programmed cell death. However, none of these mechanisms fully explain the early brain damage caused by acute CNS disease (Chen et al., 2016, 2018, 2020; Zille et al., 2017).

In this review article, we summarize the most recent advances in the effects and mechanisms of ferroptosis on the CNS, which may draw attention to its potential in clinical practice for treating EBI after acute CNS disease.

## Ferroptosis

Recently, a newly identified cell death mechanism, called ferroptosis, has been widely explored (Dixon et al., 2012). In 2012, Dixon first reported a new kind of cell apoptosis based on iron-dependent programmed cell death, which is an important molecular mechanism of death for the neurons in the central nervous system, and the incidence of neurodegenerative diseases could be significantly reduced if iron-dependent cell necrosis could be prevented. This newly discovered mechanism, ferroptosis, differs from traditional cell death processes, such as apoptosis and autophagy, which is non-apoptotic forms of iron-dependent programmed cell death. For example, ferroptosis cannot be prevented by apoptosis/autophagy-related inhibitors without intracellular calcium overload. The classical morphological characteristics of cell death are the disappearance of mitochondrial cristae and significantly narrowed mitochondria, thickening of the lipid bilayer membrane, small cell size, and reduced cell connections that lead to cell separation (Dixon et al., 2012, 2015). The biological characteristics of cell death include mainly the metabolic dysfunction of iron ions, depletion of glutathione (GSH), accumulation of iron-dependent lipid overreactive oxygen species (ROS), inhibited activity or decreased levels of glutathione peroxidase 4 (GPX4; Dixon et al., 2012; Bersuker et al., 2019). Iron ions are involved in the Fenton reaction, which produces a large number of reactive oxygen species that oxidize the lipid bilayer, causing cell damage and iron-related cell death (Dixon et al., 2012). Ferroptosis is different from the traditional mechanism of cell death in that the process is regulated by a unique variety of cell signaling pathways and genes. Although the specific regulatory network is not currently clear, abnormal metabolism involving iron ions and aberrant lipid peroxidation is the central processes in iron-related death (Stockwell et al., 2017; Green, 2019). Recent studies have also confirmed that ferroptosis is widespread in diseases of the central nervous system and is involved in the repair, aging, tumor development, cerebral hemorrhaging, and ischemia of the central nervous system (Tuo et al., 2017; Wenzel et al., 2017; Zille et al., 2017; Xie et al., 2019). Dixon reported that a mouse hippocampal slice-based training model that maintains the connections between neurons, including excitatory and inhibitory neurons, and saves microglia and astrocytes. This model represents the hippocampus *in vivo*, in which the excitability of deadly sinistral glutamic acid can cause the ferroptosis of neurons. The mechanism of ferroptosis is the same as that of induced erastin-mediated tumor necrosis, a process that can be prevented by inhibitors of ferroptosis (ferrostatin-1; Dixon et al., 2012). Zille et al. (2017) reported that the mechanisms of ferroptosis and programmed necrosis have been found in experimental cerebral hemorrhage animal models but are not dependent on apoptosis or autophagy, and blocking either ferroptosis or programmed necrosis contributes to cell survival. Wenzel et al. (2017) also confirmed that hippocampal neurons undergo ferroptosis in an animal trauma model and verified the importance of the phosphatidylethanolamine-binding protein 1 (PEBP-1)-dependent regulation mechanism of iron-related death. Therefore, the ferroptosis mechanism may be an important molecular mechanism of neuronal death, which may play an important role in EBI after acute CNS disease.

## The Mechanism of Ferroptosis

### GPX4 and Ferroptosis (Figure 1)

#### GPX4–GSH–Cysteine Axis

**Figure 1:**
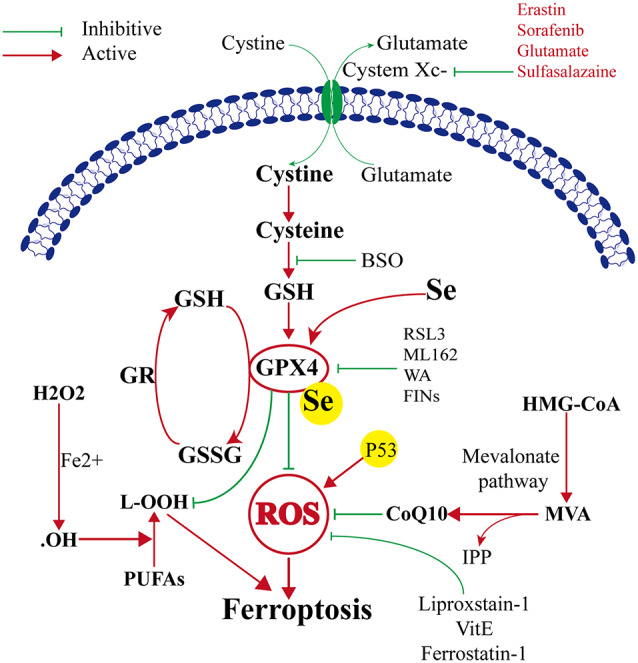
Mechanism of ferroptosis, glutathione peroxidase 4 (GPX4) glutathione (GSH) cysteine axis and mevalonate pathways.

Ferroptosis was first reported to induce iron-dependent cell death by small molecules erastin and 1S, 3R-RSL3 (RSL3; Dixon et al., 2012). These small molecules were characterized based on their ability to regulate programmed cell death in cancer cells. The classical feature of cell death after erastin or RSL3 treatment was its independence from caspase activation (Dixon et al., 2012; Wang et al., 2016). The target of erastin is System X_c_− (containing SLC3A2 and SLC7A11), which is a glutamate/cysteine antiporter in the plasma membrane of the cell. System X_c_− can transfer cysteine into the cell and transfer glutamate extracellularly. Cystine is very important because it can be converted to cysteine, which is required for the synthesis of glutathione. Glutathione is an important antioxidant that protects cells against oxidative damage. Therefore, erastin can inhibit intracellular cysteine production; thus, the level of intracellular glutathione degrades significantly, and ROS accumulate to induce ferroptosis, leading to increased cell oxidative damage (Dixon et al., 2012). GPX4 is the key protein in ferroptosis, and it needs GSH as a substrate to finish its lipid repair function. Reduced cysteine levels lead to GSH depletion, and the levels and activity of GPX4 decrease significantly, resulting in the accumulation of unrepaired lipid peroxides, lipid peroxidation, and ferroptosis (Angeli et al., 2014; Stockwell et al., 2017). Additionally, GSH is a very important component with Fe^2+^ in the labile iron pool (LIP), which binds Fe^2+^ to prevent iron oxidation. Therefore, the direct inhibition of GSH biosynthesis leads to lower steady-state intracellular GSH levels, which can trigger ferroptosis (Hider and Kong, 2011). Additionally, GPX4 can convert GSH to oxidized glutathione (GSSG), which ultimately reduces the levels of lipid hydroperoxides by converting them into alcohol or free hydrogen peroxide (Stockwell et al., 2017; Gaschler et al., 2018; Forcina and Dixon, 2019). This process can be inhibited by RSL3 (Dixon et al., 2012; Yang et al., 2014), FINO_2_ (Gaschler et al., 2018), etc. Therefore, ferroptosis can be induced by RSL3, and FINO_2_ treatment effects similar to that of inhibited GPX4 inactivation. On the other hand, selenium (Se) was the first extensively reported key regulator of GPX4 activity (Ingold et al., 2015, 2018; Angeli and Conrad, 2018; Alim et al., 2019). Alim et al. (2019) reported that pharmacological Se supplementation effectively inhibits GPX4-dependent ferroptotic death, as well as cell death induced by excitotoxicity or ER stress, and can inhibit cell death and improve cell function when administrated after hemorrhagic or ischemic stroke. Several other metabolic pathways modulate cell sensitivity to ferroptosis by regulating GPX4 (Figure 1).

#### The Mevalonate Pathway

The mevalonate pathway is also implicated in the regulation of ferroptosis and leads to the production of coenzyme Q10 (CoQ10), which is required to shuttle electrons as part of the mitochondrial electron chain. The mevalonate pathway expresses isopentenyl pyrophosphate (IPP) to enhance the translation of GPX4 by stabilizing selenocysteine-specific tRNA (Ingold et al., 2018). The ferroptosis-inducing compound FIN56 depletes CoQ10 by modulating squalene synthase activity (SQS). FIN56 also binds to and activates squalene synthase, an enzyme involved in isoprenoid biosynthesis, independent of GPX4 degradation (Shimada et al., 2016). The HMG CoA reductase inhibitor is the rate-limiting enzyme in the mevalonate pathway, possibly *via* the depletion of CoQ10 and inhibition of downstream tRNA isopentenylation (TRIT1), which is required for the biosynthesis of GPX4, thus it can induce the cell ferroptosis (Shimada et al., 2016; Viswanathan et al., 2017; Figure 1).

#### The FSP1–CoQ10–NAD (P)H Pathway

**Figure 2:**
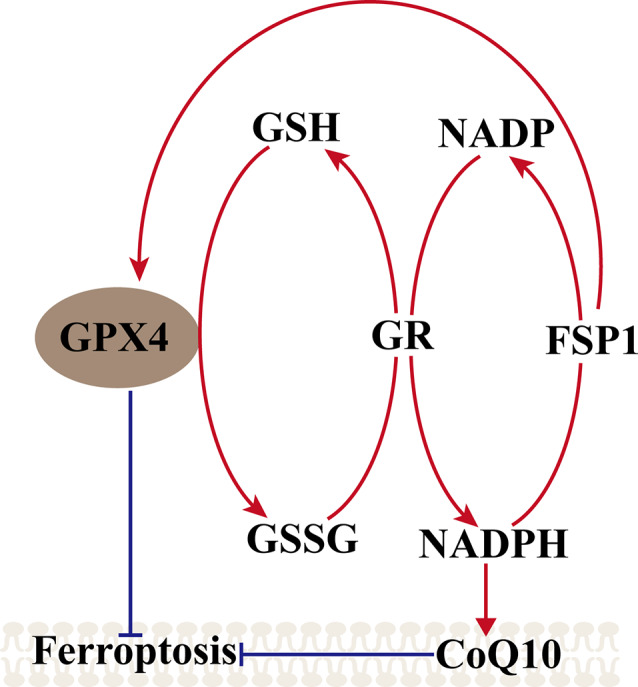
Mechanism of ferroptosis, ferroptosis suppressor protein 1 (FSP1)–coenzyme Q10 (CoQ10)–NAD (P)H pathway. GPX4 utilizes the GSH as a substrate to reduce lipid hydroperoxides and inhibited the ferroptosis, GSSG generated by GPX4 is reduced back to glutathione by GR in Nicotinamide Adenine Dinucleotide Phosphate (NADPH), ferroptosis suppressor protein 1 (FSP1) can complement the loss of GPX4, conferring protection against ferroptosis, and FSP1 catalyzes the regeneration of CoQ10 through NAD (P)H. NAD (P)H also can inhibit the ferroptosis *via* CoQ10.

Another metabolic pathway is the pentose phosphate pathway, and Nicotinamide Adenine Dinucleotide Phosphate (NADPH) also impacts ferroptosis sensitivity. NADPH is a very important reductant in the cell and is essential for balancing lipid hydroperoxide levels. Shimada et al. (2016) suggested that the level of NADPH may be a biomarker of ferroptosis sensitivity in many cancer cell lines. Recent studies found a new gene, apoptosis-inducing factor mitochondria-associated 2 (AIFM2), which was previously unrecognized as an anti-ferroptosis gene (Bersuker et al., 2019; Doll et al., 2019). Doll et al. (2019) renamed AIFM2 ferroptosis suppressor protein 1 (FSP1), which can complement the loss of GPX4, conferring protection against ferroptosis. Furthermore, the suppression of ferroptosis by FSP1 is mediated by CoQ10, and FSP1 catalyzes the regeneration of CoQ10 through NAD (P)H. Therefore, the FSP1–CoQ10–NAD (P)H pathway exists as a stand-alone parallel system that cooperates with GPX4 and glutathione to suppress phospholipid peroxidation and ferroptosis (Figure 2).

#### The NRF2/ARE–GPX4 Pathway

The transcription factor nuclear factor erythroid 2-related factor 2 (Nfe2L2, commonly referred to as Nrf2) regulates the expression of more than 250 genes and is marked by its binding site, ARE (antioxidant response element; Hayes and Dinkova-Kostova, 2017). Nrf2 is primarily maintained by Kelch-like ECH-associated protein 1 (Keap1)-mediated proteasomal degradation under non-stress conditions. Keap1 is degraded after oxidative stress is induced as it dissociates from Nrf2, allowing Nrf2 to translocate into the nucleus to initiate the transcription of antioxidant response element (ARE)-containing genes (Sun et al., 2016; Dodson et al., 2019; Kajarabille and Latunde-Dada, 2019). The Nrf2-Keap1 protein complex is a vital inhibitor of ferroptosis because of its ability to modulate the cellular antioxidant response and mitigate electrophilic or oxidative stress, proteostasis, xenobiotic/drug metabolism, iron/heme metabolism, carbohydrate, and lipid metabolism, et cetera (Dodson et al., 2019). Sun et al. (2016) reported that genetic or pharmacologic inhibition of Nrf2 expression/activity in HCC cells increased the anticancer activity of erastin and sorafenib *in vitro*, and in tumor xenograft models, Nrf2 is a key factor that determines the therapeutic response to ferroptosis-targeted therapies in HCC cells. Fan et al. (2017) reported that activation of Nrf2-Keap1 signaling upregulates xCT (also known as SLC7A11 or system Xc−) and amplifies glutamate secretion, thereby impacting the tumor microenvironment. Both these outcomes foster Nrf2 expression, and in contrast, Keap1 inhibition promotes cell resistance to ferroptosis.

**Figure 3:**
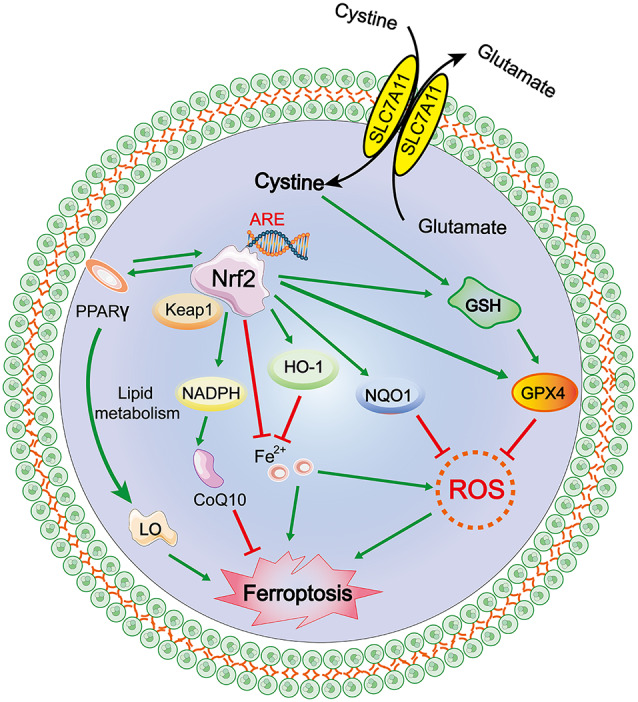
Mechanism of ferroptosis, NRF2/antioxidant response element (ARE)–GPX4 pathway.

Nrf2 activation-induced augmentation of the levels of cellular glutathione and NADPH (Ushida and Talalay, 2013), a key electron donor needed for the reduction of oxidized substrates, with GPX4 and glutathione suppress phospholipid peroxidation and ferroptosis. On the other hand, as both GPX4 and SLC7A11 are Nrf2 target genes, activated Nrf2 protects cells against hydroperoxide production and ferroptosis by upregulating the transcription of GPX4 and SLC7A11 directly (Kwak et al., 2002; Osburn et al., 2006). Shin et al. (2018) indicated that activation of the Nrf2–ARE pathway contributed to the resistance of HNC cells to GPX4 inhibition, and inhibition of this pathway reversed cell resistance to ferroptosis in HNC. Thus, the role of the NRF2/Keap1-GPX4 signaling pathway in mediating lipid peroxidation and ferroptosis is very important in terms of the established Nrf2 target genes that mitigate these pathways and the relevance of the Nrf2-lipid peroxidation-ferroptosis axis in disease (Dodson et al., 2019). Additionally, activation of Nrf2 can also promote iron storage, reduce cellular iron uptake, and limit ROS production; the specific mechanism is discussed in a subsequent section (Figure 3).

### Iron Metabolism and Ferroptosis

Iron is an essential component of the human body, as it is necessary for cellular metabolism, energy production, replication, and growth. Iron exists both as Fe^2+^ and Fe^3+^, and circulating iron exists in the form of ferric iron (Fe^3+^) by binding to transferrin. Fe^3+^ is imported into cells through the membrane protein TFR1 (transferrin receptor 1), and then, Fe^3+^ is reduced to Fe^2+^ by the activity of STEAP3 (STEAP3 metalloreductase) in endosomes (DeGregorio-Rocasolano et al., 2019). DMT1 (divalent metal transporter 1)/SLC11A2 regulates the release of Fe^2+^ from endosomes into the LIP of the cytoplasm (Ohgami et al., 2005; Xie et al., 2016; Zhou et al., 2019). Iron export is regulated by the membrane protein ferroportin (DMT1/SLC11A3), and iron is stored in ferritin, which includes ferritin light chain (FTL) and ferritin heavy chain 1 (FTH1; Xie et al., 2016; Zhou et al., 2019). Redox activity and cycling between these two states (Fe^2+^/Fe^3+^) lead to the generation of ROS through the Fenton reaction. Intracellular iron accumulation is a central biochemical event leading to ferroptosis (Bogdan et al., 2016). Ferroptosis requires transferrin and transferrin receptors to import iron from the extracellular environment, and autophagy can modulate cell sensitivity to ferroptosis through its impact on iron metabolism (Gao et al., 2015; Hou et al., 2016). The silencing of the iron metabolism master regulator IREB2 can also decrease the sensitivity of cells to ferroptosis (Dixon et al., 2012). Therefore, cellular iron metabolism, including iron uptake, export, utilization, and storage, is reprogrammed by ferroptosis (Zhou et al., 2019). Increased iron uptake and reduced iron storage may contribute to iron overload during ferroptosis, and decreased iron overload by iron chelators (e.g., deferoxamine, desferrioxamine mesylate, etc.) inhibit erastin-mediated ferroptosis, whereas supplying exogenous sources of iron enhances erastin-mediated ferroptosis (Dixon et al., 2012). Conversely, adding iron-bound transferrin or a bioavailable form of iron, but not the other divalent transition metal ions (such as Cu^2+^ or Mn^2+^), potentiated erastin-induced ferroptosis (Dixon et al., 2012; Gao et al., 2015).

**Figure 4:**
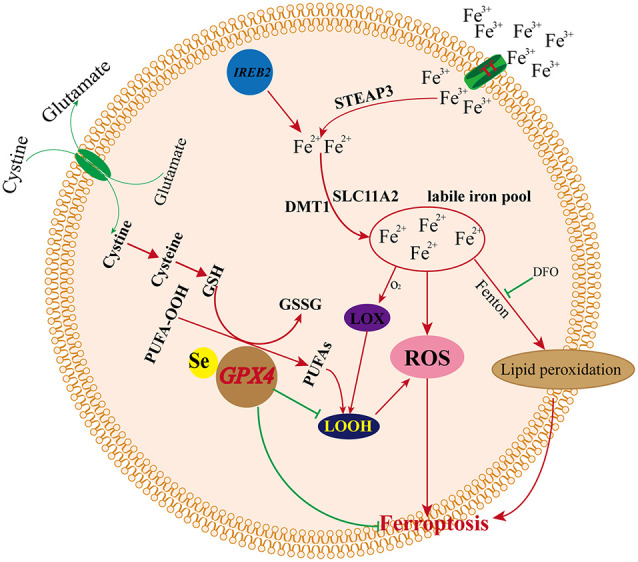
Mechanism of ferroptosis and iron metabolism.

How iron promotes ferroptosis inside the cell remains unclear. Recent studies suggest that iron promotes ferroptosis by donating electrons to oxygen to form ROS (Stockwell et al., 2017), and iron ions can follow three pathways after the iron-dependent accumulation of lipid ROS in ferroptosis: (1) two iron states (Fe^2+^/Fe^3+^) lead to the generation of ROS through the Fenton reaction; (2) ROS generated in an iron-catalyzed enzymatic manner induce lipid autoxidation; and (3) iron is also an important component in the catalytic subunit of LOX, and ROS generated from iron-dependent LOX enzymes catalyze the site-specific oxidation of PUFAs (Dixon et al., 2015). Previous studies indicated that small-molecule LOX inhibitors block cell death by depleting GSH or Gpx4, and lipophilic iron chelators may directly inactivate iron-containing enzymes that promote membrane lipid oxidation (Wang et al., 2004; Dixon et al., 2012, 2015). Thus, iron metabolism is a very important potential point of additional ferroptosis control. However, how iron regulates ferroptosis and the specified mechanism remains unclear, and more related basic research is needed to explore the relationships between iron and ferroptosis (Figure 4).

### ROS Metabolism and Ferroptosis

ROS play a fundamental role in cell and tissue injury and are generated by extracellular or intracellular stimuli (Ryter et al., 2007). Ferroptosis is generally considered a type of ROS-dependent regulated necrosis. ROS are partially reduced oxygen-containing molecules that are free radicals (HO. and RO.), hydrogen peroxide (H_2_O_2_ and ROOH), hydroxyl radicals, lipid hydroperoxides, etc. ROS can also be generated from other pathways, including those of xanthine oxidase, NADPH oxidase, cyclooxygenases, and activated neutrophils, eosinophils, and macrophages (Frey and Reed, 2012; Latunde-Dada, 2017). ROS are converted to hydrogen peroxide by superoxide dismutase (SOD) and yield the highly toxic hydroxyl radical in the presence of reduced iron (Fe^2+^) through the Fenton reaction (Wen et al., 2013). Imbalances in the rate of ROS generation and detoxification lead to oxidative stress and the consequent production of free radicals that can damage DNA, proteins, and lipids (Latunde-Dada, 2017). ROS are generated, independent of iron, by glucose and glutamine metabolism that diminishes glutathione and GPX4 levels. Dixon and Stockwell (2014) reviewed the importance of ROS and iron as initiators and mediators of cell death (ferroptosis) in many organisms and diseases, explaining the inactivation of the cystine-glutamate antiporter (SLC7A11) and glutathione depletion cause iron-dependent accumulation of ROS. Additionally, ROS can react with polyunsaturated fatty acids (PUFAs) in lipid membranes and induce lipid peroxidation. Angeli et al. (2014) and Skouta et al. (2014) reported that the execution of ferroptosis requires depletion of arachidonic acid (AA) and other PUFAs following GPX4 inactivation. On the other hand, ferroptosis is aggravated in GPX4-deficient cells, which exhibit enriched oxidized membranes containing AA, and it can enhance ferroptosis after 5-, 12-, and 15-hydroperoxyeicosatetraenoic acid (HPETE) treatment (Angeli et al., 2014). Although ROS in peroxidation can regulate ferroptosis through several pathways, the specifications of other effectors and molecular signaling events that lead to cell death by ferroptosis are unclear, and more research is needed.

## Early Brain Injury and Ferroptosis

### Ischemic Stroke

Stroke is a leading cause of death and a major cause of permanent disability, with 40% mortality within 12 months and 50% of all SAH patients becoming permanently dependent (Krishnamurthi et al., 2013). A systematic analysis for the Global Burden of Disease Study 2016 reported that, although age-standardized deaths due to stroke have been decreasing, the overall burden of stroke remains high (Johnson et al., 2019). Acute ischemic stroke is the most common form of stroke, and it leads to a worse outcome when the area of large cerebral vascular occlusion is large. Although intravenous thrombolysis and mechanical thrombectomy are good emergency clinical methods, anti-inflammation, anti-excitotoxicity, and antioxidative stress are the basis of research on the regulation of brain injury after ischemic cerebral stroke. However, more than one-half of patients fail to demonstrate clinical improvement (Yeo et al., 2013; Kikuchi et al., 2014). An increasing number of recent studies confirmed that free iron accumulates in the ipsilateral hypoxic-ischemic neonatal rat cortex (Palmer et al., 1999), total iron is increased significantly in the ischemic areas after ischemic stroke (Tuo et al., 2017), and the levels of transferrin receptors (TfRs) and iron-loaded transferrin (holo-transferrin, HTf) also increase after ischemic stroke (Park et al., 2011; DeGregorio-Rocasolano et al., 2018). All of this evidence indicates that a new cell death mechanism may play a very important role in EBI after ischemic stroke: ferroptosis (Dixon et al., 2012; Alim et al., 2019; Guan et al., 2019; Li et al., 2019; Datta et al., 2020). Inhibitors of ferroptosis, such as ferrostatins, liproxstatins, and iron chelators, can relieve brain damage and neuronal death from ischemic injury (Palmer et al., 1994; Satoh et al., 2000). Ahmad et al. (2014) reported that sesamin induces significant neuroprotection by ameliorating many signaling pathways, the level of GSH was markedly reduced and lipid peroxidation increased after ischemic stroke, and exhibited an effect similar to that in PC-12 cells in an oxygen-glucose deprivation (OGD) experimental model (Liu et al., 2017).

Although an increasing number of studies had confirmed the role of ferroptosis after ischemic stroke, these newly described mechanisms and findings provide new ideas for the treatment of ischemic stroke; however, the mechanism of iron accumulation, new cross-talk molecules, and deeper molecular pathways are unclear and need to be explored.

### Traumatic Brain Injury

TBI is among the most important public health problems; it has a significant influence on the lives of the injured individuals and family members and has a high incidence and mortality rate (Zhao and Wang, 2001; Jiang et al., 2019). TBI triggers multiple cell death pathways. Neuronal death contributes to the neurologic deficits observed after a TBI is sustained, and thus, neurons constitute a reasonable therapeutic target. Previous studies found that inhibition of apoptosis, necrosis, necroptosis, and autophagy by inhibitors (Z-VAD-FMK, BOC-D-FMK, wortmannin, and necrostatin-1) cannot relieve RSL-induced cell death. Ferroptosis is a recently described regulated cell death pathway that results from the accumulation of lipid oxidation products. Xie et al. (2019) reported that iron accumulation, dysfunctional iron metabolism, the upregulation of ferroptosis-related genes, reduced glutathione peroxidase (GPx) activity, the accumulation of lipid reactive oxygen species (ROS) after TBI, and Fer-1 significantly reduced iron deposition and neuronal degeneration while attenuating injury lesions and improving long-term motor and cognitive function. Kenny et al. (2019) indicated that depletion of glutathione in the ipsilateral cortex and accumulation of oxidized phosphatidylethanolamine baicalein can decrease ferroptosis-related phosphatidylethanolamine oxidation and improve outcomes after TBI, because of baicalein activity is related to the 15-lipoxygenase pathway. Li et al. (2019) also confirmed that baicalein exerted neuroprotective effects against posttraumatic epileptic seizures by suppressing ferroptosis and 12/15-LOX in an iron chloride (FeCl3)-induced posttraumatic epileptic seizure mouse model. The levels of GSH and GPX4 are reduced significantly after TBI (Wenzel et al., 2017; Jiang et al., 2019; Kenny et al., 2019; Li et al., 2019; Xie et al., 2019), as rich GSH and GPX4 are essential for the maintenance of redox neuro-homeostasis and for deeper exploration. Wenzel (Wenzel et al., 2017) found that the high levels of 15-HpETE-PE in the brain cortex and hippocampus after TBI, along with an increased expression of 15LO2 and decreased levels of GPX4, strongly suggest the possibility of ferroptosis. Inhibiting the ability of PEBP1/15LO complexes to form 15-HpETE-PE may lead to novel anti-ferroptosis approaches and potential neuroprotective drugs. In addition to regulating ferroptosis indicators such as desferrioxamine, vitamin E, U0126, ferrostatin-1, and liproxstatin-1 to regulate ferroptosis, Xiao et al. (2019) demonstrated that the administration of miR-212–5p significantly improved learning and spatial memory in a controlled cortical impact model, and the same result was observed in HT-22 and neuro-2a cell lines. The mechanism may reflect miR-212–5p protection against neuronal ferroptosis by targeting Ptgs2 *in vivo* and *in vitro* TBI models. This mechanism also provides us with new ideas for preventing ferroptosis.

### Intracerebral Hemorrhage and Ferroptosis

ICH is a stroke subtype associated with hypertension, moyamoya disease, arteriovenous malformations, and anticoagulant use. Acute ICH due to a large intracranial hematoma is associated with high morbidity and mortality, and craniotomy hematoma evacuation to limit secondary damage, including neuron death after ICH, is of intense interest (Hanley et al., 2019). Lysed blood, with hemoglobin, the oxidized form of iron-rich heme, is the main cause of secondary damage and neuronal death (Keep et al., 2012; Schallner et al., 2015). Recent studies and strategies have demonstrated that by limiting iron toxicity and ROS production, ferroptosis can prevent secondary damage, reduce EBI, and improve the outcome of ICH patients (Wang, 2010; Karuppagounder et al., 2016, 2018; Selim et al., 2019). The levels of GPX4 in the brain were reduced gradually, reaching the lowest level 24 h after ICH, while increased levels of GPX4 alleviated brain edema, BBB injury, neuronal dysfunction, oxidative stress, and inflammation after ICH, and an inhibitor of ferroptosis (ferrostatin-1) significantly reduced secondary brain damage (Zhang et al., 2018). Li et al. (2017) found a similar result, with ferrostatin-1 reducing lipid reactive oxygen species production and attenuating the increased expression level of PTGS2 and its gene product cyclooxygenase-2 *in vitro* and *in vivo*, suggesting that cyclooxygenase-2 is a biomarker of ferroptosis. Zille et al. (2017) also confirmed that ferroptosis and necroptosis were the main features of experimental hemorrhagic stroke *in vitro* and *in vivo* and suggested that the ferroptosis inhibitor deferoxamine may elicit significant functional recovery in some models and clinical patients of ICH. Li et al. (2018) also observed the ultrastructure of ICH-induced pathology through transmission electron microscopy, including in cells undergoing ferroptosis, necroptosis, and autophagy.

As we mentioned above, the transcription factor nuclear factor erythroid 2-related factor 2 (Nfe2L2, commonly referred to as Nrf2) has been shown to have many functions, as it can regulate more than 250 genes. Chang (Chang et al., 2014) demonstrated that (-)-epicatechin (EC), a brain-permeable flavanol that modulates redox/oxidative stress *via* the Nrf2 pathway, ameliorated neurologic deficits in ICH mice. The mechanism not only attenuated oxidative insults, increasing phase II enzyme expression by increasing the level of Nrf2, but also reduced heme oxygenase-1 induction and brain iron deposition *via* an Nrf2-independent pathway, which inhibited the activity of matrix metalloproteinase 9, reduced the level of lipocalin-2, and suppressed the expression of ferroptosis-related genes. In addition to EC, N-acetylcysteine (NAC) is a clinically approved thiol-containing redox modulatory compound that can induce postinjury neuron death and improve patient outcomes after ICH by mimicking chemical or molecular lipid peroxidation inhibitors and synergizing clinically approved prostaglandin E2 (PGE2; Karuppagounder et al., 2018). A recent study indicated that pharmacological selenium (Se) effectively inhibits GPX4-dependent ferroptosis and attenuates the cell death induced by excitotoxicity or ER stress, which is are GPX4 independent processes, *via* the coordinated activation of the transcription factors TFAP2c and Sp1 to protect neurons after hemorrhagic stroke (Alim et al., 2019). Thus, ferroptosis is a very important mechanism, and anti-ferroptosis mechanisms are potential therapeutic targets essential for future preclinical studies on ICH.

### SAH and Ferroptosis

Spontaneous subarachnoid hemorrhage (SAH) is a common cerebrovascular disease, accounting for approximately 5% of all stroke cases. The mortality in the short-term for patients after SAH is as high as 8.3–66.7%, and the mean median proportion of direct death before admission is 8.3%, with a good prognosis for only approximately 36% to 55% of these patients (Nieuwkamp et al., 2009; Chen et al., 2020). The pathological process of aneurysm rupture causing SAH and the mechanisms involved are extremely complex, and some studies have divided the pathological process into two stages: EBI and delayed brain injury (DBI; Chen et al., 2014, 2016, 2020). Regardless of the mechanism, the initiating factor in both EBI and DBI is blood entering the SAH area or brain tissue, while the lytic products of red blood cells release a large number of toxic substances to cause cerebral vasospasm and EBI, which eventually lead to secondary brain injury. Therefore, the toxic substances released by red blood cells after SAH are the initial and key factors that cause secondary brain injury (Bulters et al., 2018). Although the pathological mechanism of SAH is similar to that of ICH, the pathological mechanism of SAH is more complicated than that of ICH. Previous studies have confirmed that the Nrf2 pathway is active and confers protection after SAH and finally the evidence supporting Nrf2 upregulation has been examined for use as therapy after SAH (Zhang et al., 2019a,b; Zolnourian et al., 2019). The effects of Nrf2 upregulation are closely related to ferroptosis. Although no studies have studied SAH and ferroptosis, research exploring EBI after SAH has important potential value. We hope that more studies on the mechanisms of SAH are based on ferroptosis.

## Conclusions and Perspectives

In this review article, we have outlined our understanding of the ferroptosis pathway, mechanism of ferroptosis, important molecules directly or indirectly targeting iron metabolism and lipid peroxidation, and related regulatory mechanisms. An increasing number of early brain injuries, including ICH, ischemic stroke, and TBI, has led to research focused on ferroptosis. We also summarized the recent advances in ferroptosis in EBI after ICH, ischemic stroke, and TBI. However, many questions remain unanswered.

How is ferroptosis triggered at the molecular level in different early brain injuries and neuronal death? Should new clinical trials be initiated to test the concept of ferroptosis inhibitors as key modulators of pathological cell death during brain injury? What is the result of ferroptosis after acute CNS disease? Even though these questions have no specific answers to date, many other questions also need to be explored deeply. However, it is already clear that ferroptosis is a newly identified and important mechanism of cell death, especially in CNS disease. Anti-ferroptosis mechanisms and ferroptosis inhibitors will play an essential role in preventing EBI.

## Author Contributions

All authors listed have made a substantial, direct and intellectual contribution to the work, and approved it for publication.

## Conflict of Interest

The authors declare that the research was conducted in the absence of any commercial or financial relationships that could be construed as a potential conflict of interest.
